# Salivary Flow, Biochemical Profile and Selected Antibacterial Properties of Saliva in Menopausal Women

**DOI:** 10.3390/jcm15207934

**Published:** 2026-10-14

**Authors:** Aleksandra Ciesielska, Aida Kusiak, Magdalena Emilia Grzybowska, Barbara Kochańska, Jolanta Ochocińska, Dominika Cichońska, Dariusz Świetlik

**Affiliations:** 1 Department of Periodontology, Medical University of Gdansk, 80-210 Gdansk, Poland; aida.kusiak@gumed.edu.pl (A.K.);; 2 Department of Gynecology and Obstetrics, Medical University of Gdansk, 80-214 Gdansk, Poland; magdalena.grzybowska@gumed.edu.pl; 3 Department of Conservative Dentistry, Medical University of Gdansk, 80-210 Gdansk, Poland; 4 Department of Biostatistics and Neural Networks, Medical University of Gdansk, 80-210 Gdansk, Poland; dariusz.swietlik@gumed.edu.pl

**Keywords:** menopausal women, saliva, ionized calcium, physicochemical properties of saliva

## Abstract

**Objectives:** The aim of this study was to estimate differences in selected physicochemical and antibacterial properties of saliva between menopausal and premenopausal women. **Methods:** The study population consisted of 30 menopausal women and 22 premenopausal women assigned to the control group. Laboratory analyses included the assessment of unstimulated salivary flow rate, pH, concentration of total protein, lactoferrin, lysozyme, immunoglobulin A (IgA), inorganic phosphates, and ionized calcium. Only participants with clinically healthy periodontal tissues were included in the study. **Results:** Among menopausal women, statistically significant differences were observed in values of salivary flow rate, total protein, inorganic phosphates, and ionized calcium; however, no statistically significant differences for pH, lactoferrin, lysozyme, and IgA values were found. The unstimulated salivary flow rate parameter in the study group was significantly lower than in premenopausal women. Concentrations of total protein, inorganic phosphates, and ionized calcium in saliva of menopausal women were distinctly higher than in the control group. **Conclusions:** The results of the present study demonstrate a distinct alteration in salivary homeostasis characterized by significantly reduced salivary flow and increased concentrations of calcium, inorganic phosphate, and total protein between menopausal and premenopausal women. It suggests that menopause may predominantly affect the quantitative secretion and physicochemical composition of saliva rather than causing a generalized impairment of its antimicrobial and immune components. Longitudinal studies that control for age and other relevant factors, including menopausal duration, hormone therapy, and periodontal status, and that evaluate both salivary concentrations and individual analyte output rates are needed to clarify which salivary changes are independently associated with menopause and to determine their clinical relevance to oral health.

## 1. Introduction

Menopause is a physiological stage of female aging, defined as the permanent cessation of menstruation resulting from the depletion of ovarian follicular activity and a sustained decline in estrogen production [1,2]. Owing to increased life expectancy worldwide, women now spend approximately one-third of their lives in the postmenopausal period, making the preservation of health during this life stage an important public health priority [3,4]. While vasomotor, metabolic, skeletal, and genitourinary manifestations of menopause are well recognized, increasing evidence suggests that menopause is also associated with changes in oral health that may adversely affect quality of life, nutrition, and dental treatment outcomes [5,6].

The oral cavity represents a hormonally responsive environment. Estrogen receptors are expressed in several oral tissues, including the oral and gingival epithelium, periodontal tissues, alveolar bone, and salivary glands, providing a biological basis for the effects of estrogen on oral homeostasis. Estrogen signaling has been implicated in the regulation of epithelial integrity and maturation, extracellular matrix turnover, inflammatory and immune responses, periodontal tissue homeostasis, bone remodeling, and salivary gland function. Accordingly, the decline in circulating estrogen associated with menopause may contribute to alterations in oral epithelial and periodontal homeostasis, alveolar bone metabolism, and salivary gland physiology [7,8,9,10]. Among the oral symptoms frequently reported by peri- and postmenopausal women are xerostomia, burning mouth syndrome, dysgeusia and other disturbances of taste perception, and nonspecific oral discomfort [11,12,13]. These symptoms may negatively influence oral-health-related quality of life and compromise daily activities such as eating, swallowing, and speaking. Periodontal tissues may also be affected during the menopausal transition. Estrogen deficiency is associated with increased osteoclast activity, enhanced bone turnover, and modulation of inflammatory pathways that could increase susceptibility to periodontal tissue breakdown. Nevertheless, contemporary periodontal research recognizes menopause as one of several modifying factors that may modify periodontal health [11,14,15]. Moreover, recent systematic and scoping reviews emphasize that the prevalence and severity of oral manifestations during menopause vary considerably among studies [11,12]. Consequently, the causal relationship between menopause and specific oral disorders remains incompletely understood.

Saliva is essential for maintaining oral homeostasis through lubrication of oral tissues, buffering of acids, facilitation of mastication and swallowing, enamel remineralization, and innate and adaptive antimicrobial defense. Beyond these functions, saliva contains numerous bioactive molecules—including secretory immunoglobulin A, lactoferrin, lysozyme, histatins, defensins, mucins, cytokines, and antioxidant enzymes—that contribute to microbial regulation and maintenance of mucosal integrity [16]. Recent studies suggest that menopause may be associated not only with alterations in salivary flow rate but also with changes in selected physicochemical, protein, inflammatory, oxidative, and mineral parameters [11,17,18]. Because saliva plays a central role in maintaining mineral equilibrium within the oral cavity, alterations in electrolyte composition may influence plaque mineralization, calculus formation, enamel remineralization, and periodontal health [19].

Given the increasing number of women living for decades after menopause and the growing recognition of saliva as a valuable source of information on oral and systemic health, a better understanding of menopause-associated differences in salivary characteristics is clinically relevant. Such knowledge may contribute to a more comprehensive understanding of oral changes occurring during this stage of life and may support the development of appropriate preventive strategies. Therefore, the aim of the present study was to compare salivary flow and selected biochemical and antimicrobial parameters of saliva between menopausal and premenopausal women and to determine whether menopausal status is associated with measurable alterations in salivary characteristics.

## 2. Materials and Methods

### 2.1. Participants and Study Design

This prospective, cross-sectional, observational study was conducted to assess differences in salivary flow and selected biochemical and antimicrobial parameters between menopausal and premenopausal women.

The study group comprised 30 menopausal women aged 48–55 years who had previously undergone gynecological assessment at the Gynecological Outpatient Clinic of the Family Medicine Center, Medical University of Gdańsk, Poland. Menopausal status was established clinically based on gynecological evaluation and menstrual history, with natural menopause defined as at least 12 consecutive months of amenorrhea in the absence of another physiological or pathological cause. The participants volunteered for a follow-up examination of the periodontium and oral mucosa at the Department of Periodontology. The control group consisted of 22 premenopausal women aged 20–30 years.

The clinical examination included collection of a general medical history, including assessment of the subjective sensation of oral dryness, as well as periodontal and oral mucosa examinations. The same clinical examination protocol was applied to participants in both the menopausal and premenopausal control groups. Periodontal status was assessed by measuring probing depth (PD), interproximal clinical attachment loss (CAL), and bleeding on probing (BOP). PD and CAL were recorded using a periodontal probe graduated in 1 mm increments (PCP-UNC 15, Hu-Friedy, Chicago, IL, USA). BOP was recorded during periodontal probing and expressed as the percentage of examined sites showing bleeding. Only participants with clinically healthy periodontal tissues were included in the study. Clinical periodontal health was defined as BOP at <10% of examined sites, probing depths ≤3 mm, and no clinical attachment loss attributable to periodontitis. The oral mucosa was clinically examined in all participants for the presence of pathological lesions or other clinically detectable abnormalities.

All participants were generally healthy and were non-smokers. Women with systemic or local conditions potentially affecting the oral mucosa or salivary secretion, including diabetes mellitus and salivary gland dysfunction, were excluded. Additional exclusion criteria included hormone replacement therapy (HRT), regular use of medications, and treatment with antibiotics or corticosteroids during the preceding 6 months.

Participant recruitment and saliva collection took place between January 2024 and April 2026. The study protocol was approved by the Ethics Committee of the Medical University of Gdańsk, Poland, on 4 September 2023 (approval No. KB/341-502/2023), and the approval was subsequently extended on 15 December 2025 (approval No. KB/341-617/2025). The study was conducted in accordance with the Declaration of Helsinki of the World Medical Association.

### 2.2. Saliva Collection

Unstimulated whole saliva was collected from each participant. In premenopausal women, saliva collection was performed during the preovulatory phase of the menstrual cycle following the end of menstruation.

All samples were collected in the morning, at least 2 h after the last intake of food or beverages, into sterile silicone Corning-type tubes (Corning Incorporated, Corning, NY, USA). Unstimulated saliva was collected by expectoration without chewing or other stimulation of salivary secretion.

Following collection, the samples were clarified by centrifugation at 2000× *g* for 10 min and immediately stored at −20 °C until subsequent determination of IgA, lactoferrin, lysozyme, total protein, inorganic phosphate, and ionized calcium concentrations.

The unstimulated salivary flow rate was calculated by dividing the total volume of saliva collected over 6 min by the collection time and was expressed in mL/min.

The following reference ranges were used to classify unstimulated salivary flow:

1. 0.26–0.30 mL/min: normal unstimulated salivary flow;
2. 0.10–0.25 mL/min: reduced unstimulated salivary flow (oligosialia);
3. <0.10 mL/min: markedly reduced salivary flow (xerostomia).

Salivary pH was measured immediately after sample collection using a Fisher pH meter equipped with a glass electrode.

### 2.3. Salivary Biochemical Analysis

Unstimulated whole-saliva samples obtained from menopausal and premenopausal participants were analyzed at the biochemical laboratory of the Department of Conservative Dentistry, Medical University of Gdansk, Poland.

Salivary pH was determined using a Fisher pH meter. The concentrations of IgA, lactoferrin, and lysozyme were determined using enzyme-linked immunosorbent assays (ELISA) with commercially available kits (Sigma-Aldrich, St. Louis, MO, USA) after centrifugation of the saliva samples at 10,000× *g* for 10 min.

Total protein concentration was determined using the Lowry method [20]. This assay is based on the reaction of peptide bonds and amino acid residues, particularly tyrosine, with the Folin–Ciocalteu reagent, resulting in the formation of colored reaction products. Absorbance was measured at 750 nm.

Inorganic phosphate concentration was determined using a direct phosphomolybdate method based on the method of Daly and Ertingshausen and modified by Wagner et al. [21]. In this method, the reduction step of phosphomolybdate is omitted, and the amount of phosphomolybdate formed is measured directly at 340 nm. The measured absorbance is proportional to the concentration of inorganic phosphorus in the sample.

For the assay, the reagent was added to the saliva sample at a ratio of 1:50. Following incubation for 6 min, absorbance was measured at 340 nm, and phosphorus concentration was calculated from a standard curve. An Alfa Diagnostics reagent (pH < 1) containing ammonium molybdate (0.8 mmol/L), sulfuric acid (430 mmol/L), and sodium chloride (77 mmol/L) was used. An Alfa Diagnostics phosphorus standard with a concentration of 1.61 mmol/L was used to construct the standard curve.

Ionized calcium concentration was determined using the Arsenazo III method [22]. This method is based on the binding of calcium ions to the metallochromogen Arsenazo III, resulting in the formation of a colored complex. The intensity of the color, measured spectrophotometrically at 650 nm, is proportional to the calcium concentration in the sample.

An Alpha Diagnostics reagent (pH 6.75) containing Arsenazo III (0.2 mmol/L) and imidazole buffer (100 mmol/L) was used. A ready-to-use Alpha Diagnostics calcium standard containing calcium at a concentration of 2.5 mmol/L was used to construct the standard curve. The reagent was added to each saliva sample at a ratio of 1:50 and incubated for 1 min, after which absorbance was measured at 650 nm. Calcium concentration was subsequently calculated from the standard curve.

### 2.4. Statistical Analysis

Statistical analyses were performed using Statistica (TIBCO Software Inc., San Ramon, CA, USA; version 13.3, 2017); http://statistica.io, accessed on 4 September 2026) and Microsoft Excel for Microsoft 365.

Continuous variables were summarized as the mean and standard deviation (SD) for normally distributed data and as the median and range (minimum–maximum) for non-normally distributed data. Where appropriate, 95% confidence intervals (CIs) were also calculated. Categorical variables were expressed as absolute numbers and percentages.

The distribution of continuous variables was assessed using the Shapiro–Wilk test, and homogeneity of variance was evaluated using Levene’s test.

Comparisons between two independent groups were performed using Student’s *t*-test for variables satisfying parametric assumptions and the Mann–Whitney U test for non-normally distributed variables. Comparisons involving more than two groups were performed using one-way analysis of variance (ANOVA) or the Kruskal–Wallis test, as appropriate. When a statistically significant overall difference was identified, appropriate post hoc analyses were performed.

Associations between continuous variables were evaluated using correlation analysis. Pearson’s correlation coefficient was applied when parametric assumptions were satisfied, whereas Spearman’s rank correlation coefficient was used for non-parametric data. All statistical tests were two-sided, and a *p*-value < 0.05 was considered statistically significant.

## 3. Results

Table 1 presents the values of pH, salivary flow rate, lactoferrin, lysozyme, IgA, total protein, inorganic phosphates, and ionized calcium concentration in unstimulated saliva among menopausal women and the control group.

**Figure 1:**
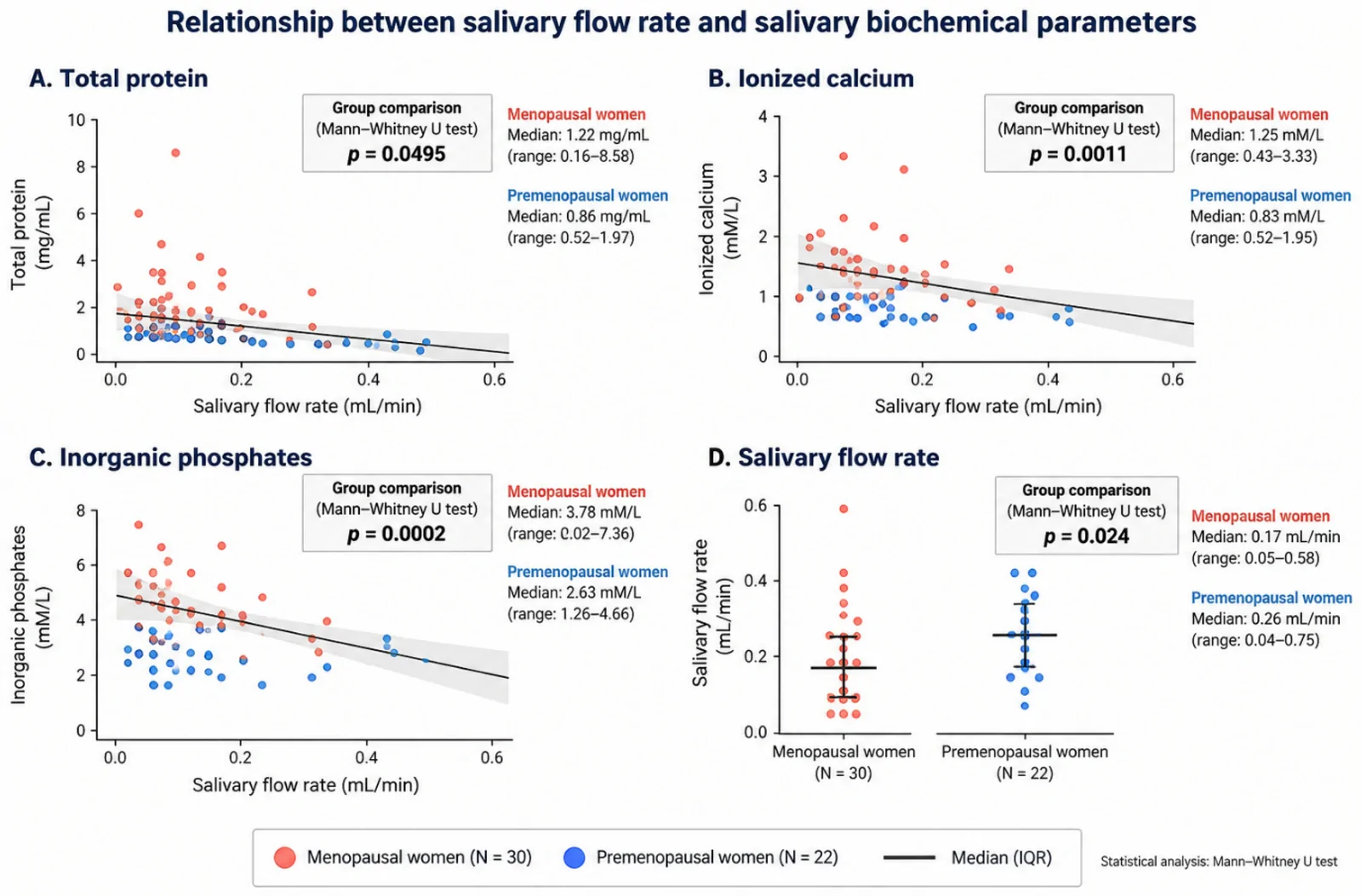
Relationship between unstimulated salivary flow rate and salivary biochemical parameters in menopausal women and the control group.

The unstimulated whole salivary flow rate was significantly lower in menopausal women than in premenopausal controls. In menopausal women, the mean salivary flow rate was 0.21 ± 0.13 mL/min (range: 0.05–0.58 mL/min; median: 0.17 mL/min), compared with 0.30 ± 0.17 mL/min (range: 0.04–0.75 mL/min; median: 0.26 mL/min) in the control group (Figure 1).

In the group of menopausal women, Pearson’s correlation analysis was performed to assess the associations between salivary flow rate and the other analyzed parameters. A very strong positive correlation was observed between salivary flow rate and saliva volume (r = 0.99, *p* < 0.0001). No statistically significant correlations were found between salivary flow rate and pH, total protein, lactoferrin, lysozyme, IgA, or calcium concentrations (all *p* > 0.05). A moderate negative correlation was observed between salivary flow rate and phosphate concentration (r = −0.34); however, this association did not reach statistical significance (*p* = 0.0645–0.0646).

A statistically significant difference in salivary total protein concentration was also observed between the groups, with higher concentrations in menopausal women. The mean total protein concentration was 2.05 ± 2.05 mg/mL (range: 0.16–8.58 mg/mL; median: 1.22 mg/mL) in menopausal women and 1.01 ± 0.44 mg/mL (range: 0.52–1.97 mg/mL; median: 0.86 mg/mL) in premenopausal controls (Figure 1).

Salivary ionized calcium concentration was significantly higher in menopausal women than in premenopausal controls. The mean ionized calcium concentration was 1.28 ± 0.54 mmol/L (range: 0.43–3.33 mmol/L; median: 1.25 mmol/L) in menopausal women and 0.90 ± 0.33 mmol/L (range: 0.52–1.95 mmol/L; median: 0.83 mmol/L) in the control group (Figure 1).

Similarly, salivary inorganic phosphate concentration was significantly higher in menopausal women. The mean concentration was 4.02 ± 1.37 mmol/L (range: 2.02–7.36 mmol/L; median: 3.78 mmol/L) in menopausal women and 2.69 ± 0.90 mmol/L (range: 1.26–4.66 mmol/L; median: 2.63 mmol/L) in premenopausal controls (Figure 1).

The mean salivary pH was 6.27 ± 0.69 (range: 4.50–8.00; median: 6.10) in menopausal women and 6.05 ± 0.55 (range: 4.50–7.00; median: 6.10) in premenopausal controls. The difference between the groups was not statistically significant.

The mean salivary lactoferrin concentration was 6.09 ± 5.70 µg/mL (range: 1.10–23.28 µg/mL; median: 4.00 µg/mL) in menopausal women and 4.64 ± 4.26 µg/mL (range: 0.56–17.00 µg/mL; median: 2.85 µg/mL) in premenopausal controls. Although the mean concentration was numerically higher in menopausal women, the difference was not statistically significant.

No statistically significant difference in salivary lysozyme concentration was observed between the groups. The mean lysozyme concentration was 3.20 ± 4.78 µg/mL (range: 0.21–20.03 µg/mL; median: 1.33 µg/mL) in menopausal women and 2.37 ± 3.27 µg/mL (range: 0.19–13.97 µg/mL; median: 1.00 µg/mL) in premenopausal controls.

The mean salivary IgA concentration was 603.19 ± 521.90 µg/mL (range: 71.00–2579.02 µg/mL; median: 401.09 µg/mL) in menopausal women and 425.85 ± 174.39 µg/mL (range: 116.76–721.83 µg/mL; median: 424.07 µg/mL) in premenopausal controls. Although the mean IgA concentration was numerically higher in menopausal women, the difference between the groups was not statistically significant.

All participants included in the study had clinically healthy periodontal tissues, in accordance with the predefined inclusion criteria. No pathological oral mucosal lesions were identified during the clinical examination in either the menopausal or premenopausal control groups.

## 4. Discussion

Saliva constitutes a fundamental component of the oral defense system and plays a central role in maintaining oral homeostasis. Adequate salivary secretion provides continuous lubrication and protection of the oral mucosa and dental surfaces and contributes to oral clearance, acid buffering, mineral homeostasis, enamel remineralization, antimicrobial defense, wound healing, and preservation of mucosal integrity [16,23]. Salivary flow is also essential for several physiological functions that directly affect oral-health-related quality of life. The fluid phase of saliva dissolves and transports tastants to receptors within the taste buds, while its lubricating properties facilitate mastication, bolus formation, swallowing, and speech [23]. Saliva contributes to the maintenance of a stable oral ecosystem by influencing microbial adhesion, aggregation, clearance, and the physicochemical conditions of microbial growth. Consequently, both the quantity and composition of saliva are important determinants of the ecological balance between the host and the oral microbiota [16,24]. Clinically, chronic hyposalivation has been associated with an increased susceptibility to dental caries, oral candidiasis, mucosal irritation and ulceration, halitosis, dysgeusia, difficulties with mastication and swallowing, and reduced tolerance of removable dental prostheses [23]. These consequences illustrate that salivary gland dysfunction may affect not only oral disease risk but also nutrition, communication, comfort, and overall quality of life [25].

Oral dryness is among the oral complaints frequently reported during the menopausal and postmenopausal periods, whereas evidence for a consistent reduction in objectively measured salivary flow is less uniform [12,13]. In the present study, menopausal women exhibited a significantly lower unstimulated salivary flow rate than premenopausal women, with values corresponding to reduced unstimulated salivary secretion. This finding is consistent with several clinical studies reporting lower salivary flow in menopausal or postmenopausal women (Foglio-Bonda et al.; Cydejko et al.; Tjahajawati et al.; Gaikwad et al. [17,26,27,28]). In a recent study, Tjahajawati et al. [17] observed a significantly lower unstimulated salivary flow rate in postmenopausal women than in premenopausal women, even after adjustment for age, body mass index, and blood pressure. The postmenopausal group also showed a significantly higher prevalence of several oral findings and symptoms, including plaque, calculus, caries, gingival recession, tooth mobility, xerostomia, and oral burning. These observations suggest that reduced salivary secretion during the postmenopausal period may coexist with a less favorable oral environment, although causal relationships cannot be inferred from cross-sectional findings. Menopausal duration may represent an additional modifying factor. Krupa et al. [29], in a study of 327 postmenopausal women, demonstrated a significant association between duration since menopause and salivary flow rate, whereas associations with tooth loss and oral-health-related quality of life were less evident. This finding suggests that years since menopause should be considered when evaluating salivary function and may partly contribute to variability among studies. If menopausal duration is not accounted for, women at different stages of the postmenopausal period may be considered together despite potentially differing cumulative hormonal and age-related influences on salivary gland function.

The biological mechanisms underlying oral dryness during the menopausal transition remain incompletely understood and are likely multifactorial. The marked decline in circulating estrogen concentrations accompanying menopause may influence salivary gland structure and function, epithelial homeostasis, inflammatory signaling, and mechanisms involved in fluid and electrolyte secretion [11]. Experimental evidence provides biological support for this relationship, suggesting that estrogen deficiency may promote inflammatory and structural alterations within the salivary glands and impair secretory function. Recent experimental work has further demonstrated interactions among estrogen deficiency, metabolic disturbances, salivary gland inflammation, and hyposalivation, reinforcing the concept that endocrine and metabolic factors may act synergistically rather than independently [9]. Nevertheless, current clinical evidence does not support a simple causal model in which declining estrogen concentrations invariably result in reduced salivary flow. Studies comparing premenopausal and postmenopausal women have yielded heterogeneous findings, and subjective xerostomia does not necessarily correspond to objectively measured hyposalivation (Butera et al.; Boonyoung et al. [11,30]). This variability may reflect differences in age, duration since menopause, systemic and metabolic health, menopausal hormone therapy, hydration, psychological factors, smoking, oral and periodontal status, and particularly the use of medications with xerogenic effects [12,17,29,30].

Furthermore, menopause-associated alterations may extend beyond salivary quantity. Changes in salivary pH, buffering capacity, electrolyte and mineral concentrations, and protein composition have been described in menopausal women. Such compositional changes could theoretically influence oral microbial ecology, enamel demineralization and remineralization, calculus formation, mucosal defense, and periodontal homeostasis even in the absence of marked reductions in total salivary flow. Saliva should therefore be evaluated as a complex biological fluid rather than exclusively in terms of its secretion rate [11,16,17,18,19,27].

In the present study, salivary total protein concentration was significantly higher in menopausal women than in premenopausal controls. This finding should be interpreted in conjunction with the significantly lower salivary flow rate observed in the menopausal group. Although evidence regarding total salivary protein concentration during menopause remains limited and heterogeneous, recent literature suggests that the menopausal transition may be associated with qualitative as well as quantitative alterations in saliva, with individual salivary constituents showing variable patterns of change [11,13]. Importantly, an increase in protein concentration does not necessarily indicate enhanced protein secretion by the salivary glands. Because salivary concentrations are strongly influenced by fluid secretion, a reduction in salivary flow may result in a relative concentration of protein constituents within a smaller volume of saliva. Therefore, the increased total protein concentration observed in the menopausal group may, at least partly, reflect reduced salivary water secretion rather than increased glandular protein output. Some studies regarding the topic of burning mouth syndrome (BMS), which disproportionately affects menopausal populations, have reported no significant differences in total salivary protein concentration or salivary flow between BMS patients and controls, despite alterations in individual salivary components, suggesting that changes in specific proteins do not necessarily translate into changes in total protein concentration [11,31,32].

Data concerning alterations in salivary inorganic phosphate concentration during menopause remain limited and inconsistent. In the present study, salivary inorganic phosphate concentration was significantly higher in menopausal women than in premenopausal controls. However, in a recent clinical study comparing premenopausal and postmenopausal women, Tjahajawati et al. [17] found no significant difference in salivary phosphate concentrations between the groups, despite evaluating salivary flow, pH, phosphate levels, and dietary phosphorus intake. In contrast, more recent research examining salivary mineral and bone-related biomarkers across pre-, peri-, and postmenopausal stages has provided evidence of menopause-associated differences involving salivary calcium and phosphorus (Mathangi et al. [18]). These discrepancies indicate that menopausal status alone may not be a major determinant of salivary phosphate concentration. Interpretation of salivary phosphate levels is complicated by several physiological and methodological factors. Phosphate concentration depends on salivary gland origin, flow rate, stimulation, circadian variation, dietary intake, oral pH, systemic mineral metabolism, and potentially periodontal and dental status. In addition, salivary calcium and phosphate jointly contribute to the degree of saturation with respect to hydroxyapatite and other calcium phosphate phases and therefore participate in enamel demineralization and remineralization as well as dental calculus formation. Consequently, changes in salivary phosphate concentration should be interpreted together with salivary flow rate, pH, calcium concentration, and relevant systemic and oral-health variables rather than as an isolated marker of menopausal status [19]. The scarcity of contemporary studies specifically investigating salivary inorganic phosphate in menopausal women highlights an important gap in the literature. Further studies using standardized saliva collection and analytical procedures and controlling for age, diet, medication use, menopausal duration, hormone therapy, bone mineral status, and periodontal condition are required to determine whether menopause independently influences salivary phosphate homeostasis.

Salivary ionized calcium concentration was also significantly higher in menopausal women than in premenopausal controls in the present study. This observation is consistent with literature suggesting that the menopausal transition may be accompanied by alterations in salivary mineral composition. Recent clinical investigations have reported increased salivary calcium concentrations in postmenopausal women, while systematic and narrative reviews have discussed the potential association between reduced estrogen levels, altered bone mineral metabolism, and elevated salivary calcium [14,18,19,27]. A recent case–control study by Tripthi et al. [33] further demonstrated the clinical relevance of evaluating salivary calcium together with salivary flow and oral dryness in postmenopausal women. Several mechanisms may contribute to these observations. The decline in estrogen after menopause is associated with increased bone turnover and alterations in bone mineral metabolism, providing a biologically plausible link between menopausal status and changes in salivary mineral composition. However, salivary calcium concentration is determined not only by systemic calcium metabolism but also by salivary gland function, salivary flow, pH, dietary calcium intake, medication use, periodontal status, and local oral conditions. Consequently, elevated salivary calcium should not be interpreted solely as a direct consequence of estrogen deficiency [19]. From a clinical perspective, increased salivary calcium may influence the oral mineral environment. Calcium, together with phosphate and salivary pH, determines the degree of saturation of saliva with respect to hydroxyapatite and other calcium phosphate phases. Higher calcium concentrations may therefore promote remineralization under appropriate conditions but may also facilitate mineralization of dental plaque and calculus formation [19,34]. The potential consequences of altered salivary calcium during menopause should consequently be interpreted in conjunction with phosphate concentration, salivary pH, flow rate, periodontal condition, and calculus accumulation.

An important consideration in interpreting the present findings is the relationship between salivary flow rate and analyte concentration. Menopausal women in the present study exhibited a significantly lower unstimulated salivary flow rate together with higher concentrations of total protein, inorganic phosphate, and ionized calcium. Because the concentration of salivary constituents may be influenced by the volume of fluid secreted, higher concentrations do not necessarily indicate increased glandular secretion of these components. Reduced salivary flow may, at least partly, contribute to higher measured concentrations by reducing the volume in which salivary constituents are distributed. Therefore, the observed increases in calcium, phosphate, and total protein concentrations should be interpreted as differences in salivary concentration rather than as evidence of increased secretory output.

In our study of saliva in menopausal women, no statistically significant differences were found in the levels of pH, lactoferrin, lysozyme, and IgA, although these parameters showed a tendency toward higher values compared to the premenopausal group. The present findings should also be considered in relation to our previous pilot study by Cydejko et al. [27]. Importantly, the participants included in the present investigation constituted a new independent cohort and did not overlap with those included in the previous study. The earlier study was designed as a preliminary investigation and included a relatively small sample of 9 menopausal and 15 premenopausal women. In that study, differences in salivary antimicrobial components were observed, with the between-group difference in lysozyme concentration reaching statistical significance. The discrepancy between the two studies may reflect biological variability in salivary antimicrobial components as well as differences in sample composition and the limited sample size of the previous pilot study.

The numerical tendency toward higher concentrations of lactoferrin, lysozyme, and IgA observed in the menopausal group may have several possible explanations. These molecules constitute important components of innate and adaptive oral defense, and their salivary concentrations may be influenced by local microbial exposure, mucosal immune activity, and periodontal inflammation. Lactoferrin and lysozyme participate predominantly in innate antimicrobial defense, whereas secretory IgA represents a major component of adaptive mucosal immunity and contributes to immune exclusion, including limitation of microbial adherence and colonization at mucosal surfaces [16,35]. The numerical tendency toward higher lactoferrin, lysozyme, and IgA concentrations observed in the menopausal group may indicate subtle alterations in oral immune homeostasis. One possible explanation is an adaptive response to changes in the local microbial or inflammatory environment accompanying the menopausal transition [11,13,36]. Given the absence of statistical significance, these findings should be interpreted cautiously and cannot be considered evidence of increased salivary antimicrobial activity after menopause. Furthermore, the present study measured the concentrations of selected antimicrobial components rather than their functional antimicrobial activity. The observed numerical differences may warrant further investigation in larger cohorts, particularly using approaches that consider salivary flow and periodontal status and distinguish salivary concentration from total analyte output. The tendency toward higher concentrations of antimicrobial components in the menopausal group raises the possibility of subtle changes in oral immune homeostasis that may not have been detectable with the present sample size. In addition, periodontal status should be considered because local inflammation and neutrophil recruitment may particularly influence salivary lactoferrin and lysozyme levels [37,38,39]. In the present study, periodontal status was clinically assessed, and only participants with clinically healthy periodontal tissues were included, thereby reducing the potential influence of active periodontal inflammation on the measured antimicrobial parameters. Nevertheless, other local and systemic factors may have contributed to the observed variability.

Several limitations of the present study should be considered when interpreting these findings. First, the relatively small sample size may have limited the statistical power to detect subtle between-group differences, particularly for salivary antimicrobial parameters. Second, the substantial age difference between the menopausal (48–55 years) and premenopausal (20–30 years) groups represents an important potential confounding factor. Chronological aging may independently influence salivary gland function, salivary flow, and salivary composition. Because age and menopausal status were closely associated in the present study population, their independent effects could not be reliably distinguished. Periodontal status represents another potentially important determinant of salivary composition, particularly with regard to inflammatory and antimicrobial components. To reduce this source of variability, periodontal status was clinically assessed, and only women with healthy periodontal tissues were included in the study. Nevertheless, residual effects of other unmeasured oral, behavioral, and systemic factors cannot be excluded. Longitudinal studies following women across the menopausal transition would be particularly valuable for distinguishing changes associated with menopause from those related to chronological aging.

## 5. Conclusions

The results of the present study demonstrate a distinct alteration in salivary homeostasis characterized by significantly reduced salivary flow and increased concentrations of calcium, inorganic phosphate, and total protein between menopausal and premenopausal women. In contrast, salivary pH and the concentrations of the antimicrobial and immune defense components lactoferrin, lysozyme, and IgA were not significantly different, although these parameters tended to be higher in the menopausal group. This suggests that menopause may predominantly affect the quantitative secretion and physicochemical composition of saliva rather than causing a generalized impairment of its antimicrobial and immune components. Overall, our study supports the concept that menopause is associated with a quantitative and qualitative remodeling of the salivary parameters. Such changes may modify oral mineral balance and protective functions and could contribute to the altered oral health conditions observed in menopausal women. Longitudinal studies that control for age and other relevant factors, including menopausal duration, hormone therapy, and periodontal status, and that evaluate both salivary concentrations and individual analyte output rates are needed to clarify which salivary changes are independently associated with menopause and to determine their clinical relevance to oral health.

## Figures and Tables

**Table 1:** Mean values of unstimulated saliva pH, salivary flow rate, lactoferrin, lysozyme, IgA, total protein, inorganic phosphates, and ionized calcium concentration in menopausal women and the control group.

		Study Group (*n* = 30)	Control Group (*n* = 22)	*p*-Value
**pH**				0.3134 ^1^
	Mean ± SD	6.27 ± 0.69	6.05 ± 0.55	
	Range	4.5–8.0	4.5–7.0	
	Me	6.1	6.1	
**Salivary flow rate [mL/min]**				0.0240 ^1,^ *
	Mean ± SD	0.21 ± 0.13	0.30 ± 0.17	
	Range	0.05–0.58	0.04–0.75	
	Me	0.17	0.26	
**Lactoferrin [µg/mL]**				0.3434 ^1^
	Mean ± SD	6.09 ± 5.70	4.64 ± 4.26	
	Range	1.10–23.28	0.56–17.00	
	Me	4.00	2.85	
**Lysozyme [µg/mL]**				0.7388 ^1^
	Mean ± SD	3.20 ± 4.78	2.37 ± 3.27	
	Range	0.21–20.03	0.19–13.97	
	Me	1.33	1.00	
**IgA [µg/mL]**				0.5596 ^1^
	Mean ± SD	603.19 ± 521.90	425.85 ± 174.39	
	Range	71.00–2579.02	116.76–721.83	
	Me	401.09	424.07	
**Total protein [mg/mL]**				0.0495 ^1,^ *
	Mean ± SD	2.05 ± 2.05	1.01 ± 0.44	
	Range	0.16–8.58	0.52–1.97	
	Me	1.22	0.86	
**Inorganic phosphates [mM/L]**				0.0002 ^1,^ *
	Mean ± SD	4.02 ± 1.37	2,69 ± 0.90	
	Range	2.02–7,36	1.26–4.66	
	Me	3.78	2.63	
**Ionized calcium [mM/L]**				0.0011 ^1,^ *
	Mean ± SD	1.28 ± 0.54	0.9 ± 0.33	
	Range	0.43–3.33	0.52–1.95	
	Me	1.25	0.83	

Legend: ^1^ Mann–Whitney U test, * *p* < 0.05. Mean—mean values; SD—standard deviation; Me—median.

## Data Availability

The datasets generated and/or analyzed during the current study are available from the corresponding author upon reasonable request. The data are not publicly available due to ethical and privacy restrictions.
